# *In vitro* and *in vivo* antioxidant potentials of *Alchornea floribunda* leaf extract, fractions and isolated bioactive compounds

**Published:** 2017

**Authors:** Daniel Lotanna Ajaghaku, Okechukwu Obasi, Blessing Ogechukwu Umeokoli, Peter Ogbuatu, Chukwuemeka Sylvester Nworu, Emmanuel Emeka Ilodigwe, Festus Basden Chiedu Okoye

**Affiliations:** 1 *Department of Pharmacology and Toxicology, Faculty of Pharmaceutical Sciences, Nnamdi Azikiwe University, Awka, Anambra State, Nigeria*; 2 *Department of Pharmaceutical and medicinal Chemistry, Faculty of Pharmaceutical Sciences, Nnamdi Azikiwe University, Awka, Anambra State, Nigeria*; 3 *Department of Pharmacology and Toxicology, Faculty of Pharmaceutical Sciences, University of Nigeria, Nsukka, Enugu State, Nigeria*

**Keywords:** Alchornea floribunda, Antioxidant activity, Flavans, Flavanone

## Abstract

**Objective:**

*Alchornea floribunda* leaves are widely used in ethnomedicine for the management of immuno-inflammatory disorders. We investigated the *in vivo* and *in vitro* antioxidant activity of the leaf extract, fractions and isolated compounds of *A. floribunda*.

**Materials and Methods:**

The ethyl acetate fraction of the methanol leaf extract was subjected to several chromatographic separations to isolate compounds 1-4. The structures of the isolated compounds were elucidated by a combination of 1D and 2D NMR and mass spectrometry. Oxidative stress was induced with carbon tetrachloride (CCl_4_). Further analysis on the isolated phenolic compounds were done using 2,2-diphenyl-1-picrylhydrazyl (DPPH), ferric reducing antioxidant power (FRAP) and hydrogen peroxide scavenging activity tests.

**Results:**

The ethyl acetate fraction at 200 mg/kg produced significant (p<0.05) elevations of catalase enzyme activity and a significant (p<0.05) reduction in serum malondialdehyde. The chemical investigation of the ethyl acetate fraction led to the isolation of three flavans, (-) cathechin (1), (-) epicathechin (2), (+) epicathechin (3) and a flavanone, 2R, 3R dihydroquercitin (4). In hydrogen peroxide scavenging assay, (-) epicathechin exhibited an EC_50 _value of 8 μg/ml, similar to the standard ascorbic acid (EC_50_ = 8 μg/ml). (-) epicathechin showed scavenging of DPPH radical with EC_50 _value of 19 μg/ml while in the FRAP assay, it had EC_50 _value of 46 μg/ml which was lower than that of the standard, ascobic acid (EC_50 _= 66 μg/ml).

**Conclusion:**

The medicinal uses of *A. floribunda *may be due to the antioxidant activities of its phenolic compounds.

## Introduction

Oxidative stress has been implicated in the development and progression of many diseases such as inflammation, cancer, coronary heart disease, atherosclerosis and Alzheimer’s disease (Soobrattee et al., 2006 ▶; Jones, 2008 ▶). The usefulness of antioxidants in the treatment and management of human diseases therefore stems from their abilities to regulate oxidative stress (Kelly, 1998 ▶). The use of synthetic antioxidants has been associated with numerous side effects (Sasaki et al., 2002 ▶) and this has necessitated further search for alternative sources of antioxidants such as natural products. Although there is limited scientific validation of the safety of natural products, it is generally accepted that natural products are safer compared to synthetic ones (Vongtau et al., 2005; Oluyemi et al., 2007 ▶). 

Plants have been identified as a huge source of structurally diverse and important antioxidant compounds with potentials for development into novel therapeutic molecules (Rout et al., 2009 ▶). *Alchornea floribunda *(*A. floribunda*) is a branched monoecious shrub widely distributed in Africa. It has been used widely as a traditional treatment for a wide range of health conditions including hepatitis, wounds, ringworm, eczema, pains in the heart, and a variety of infections and inflammatory disorders, as an antidote to poison and for general body healing (Okoye and Osadebe 2009 ▶; Noundou et al., 2014 ▶). 

In our previous works, we investigated the leaves of this plant for their anti-inflammatory activity and observed that topical and systemic anti-inflammatory activity was associated with the presence of some membrane stabilizing stigmastane steroids and a flavonoid glycoside (Okoye and Osadebe 2010 ▶; Okoye et al., 2010 ▶, 2011). In an effort to investigate the possible underlying mechanisms of the pharmacological activities of this plant material, we have evaluated the phenolic rich fractions of the methanol leaf extract for *in vivo *and *in vitro *anti-oxidant activity. It should be noted that the role of oxidative stress in the pathophysiology of some of the disease states which have been managed in ethnomedicine with the extracts of *A. floribunda *leaves is well documented (Di et al., 2010 ▶; Martin et al., 2010 ▶). Therefore, we speculate that the inhibition of oxidative stress may be an important mechanism of action of the extracts of this plant material.

## Materials and Methods

**Collection of Plant Materials**

The leaves of *A. floribunda* were collected in December 2010 from Oba Nsukka Enugu State, Nigeria, with the help of a taxonomist, Mr. Alfred Ozioko of Bioresources Development and Conservation Program, Nsukka, Enugu State, Nigeria, who also authenticated the plant material. A voucher specimen has been deposited at the herbarium of the Department of Pharmacognosy, University of Nigeria, Nsukka, Enugu State, under the herbarium number 06/0085. The leaves were air-dried for 10 days and pulverized. 

**Extraction, isolation and purification of active compounds**

About 500 g of pulverized dried leaves of *A. floribunda* were extracted with 5 l of MeOH for 7 days at room temperature (25°C) and the extract was concentrated *in vacuo* with a rotary evaporator to obtain a dark green semisolid. The dried extract was reconstituted in 20 ml of methanol and the dispersion made up to 200 ml with water, sonicated for about 10 min and subsequently, partitioned successively against hexane (750 ml × 3), ethyl acetate (750 ml × 3) and n-butanol (500 ml × 2). All fractions were taken to dryness using rotary evaporator. About 5 g of ethyl acetate fraction was subjected to VLC (siliga gel 500 g, sintered funnel 5 l) eluting with 500 ml each of hexane:ethyl acetate (90:10, 70:30, 50:50, 30:70, 10:90, and 0:100) and dichloromethane:MeOH (90:10, 80:20, 60:40, 40:60, 20:80, and 0:100) resulting in 12 pooled fractions EF1 to EF12. About 200 mg of the sub-fraction EF4 (eluted with dichloromethane:MeOH 3:7) was subjected to Sephadex LH-20 (3X60) separation using dichloromethane:MeOH 1:1 and finally, purified by semi-preparative reverse phase HPLC to obtain compounds **1 **(10 mg), **2 **(10.0 mg), **3 **(11 mg), **4** (12.0 mg).

**Phytochemical test**

Qualitative determination of secondary metabolites in the extract and fractions were carried out using the methods described by Odebiyi and Sofowora, (1978) ▶ and Trease and Evans, (2002).

**Estimation of total phenolic content**

The total phenolic content of the extract and fractions were determined using the method described by Kim et al. (2003) ▶ with modification. Here, 1 ml of the extract/fractions (0.1 mg/ml) was mixed with 0.2 ml of Folin-Ciocalteu’s phenol reagent. After 5 min, 1 ml of 7.6% Na_2_CO_3_ solution was added to the mixture followed by the addition of 2 ml of deionised distilled water. The mixtures (in triplicate) were incubated at 40^o^C for 30 min and the absorbance was read at 760 nm. The total phenolic content was determined from extrapolation of calibration curve which was made using gallic acid solution and expressed as milligrams of gallic acid equivalents (GAE) per gram of the extract/fractions.

**Structural elucidation of isolated compounds**

NMR spectra (^1^H, ^13^C, DEPT, HMQC and HMBC) were recorded with Bruker ARX 500 NMR. MS (ESI) was obtained with Finnigan LCQ Deca mass spectrometer. Analytical HPLC was carried out with a Dionex P580 HPLC system coupled with a photodiode array detector (UVD340S). Routine detection was at 235, 254, 280 and 354 nm. The separation column (125 × 4 mm, length × internal diameter) was prefilled with Eurospher-10 C18 (Knauer, Germany), and a linear gradient of nanopure water (adjusted to pH 2 by addition of formic acid) and methanol was used as eluent. Silica gel 60 F254 (layer thickness 0.2 mm, E. Merck, Darmstadt, Germany) was used for analytical TLC. Compounds were detected under UV absorbance at 254 nm (fluorescence absorption) and 366 nm (fluorescence). Vacuum Liquid Chromatography (VLC) was carried out with silica gel (230-400 mesh, Merk), Sephadex LH-20 (25−100 μm mesh size, Merk) was used for CC while TLC was performed on silica gel G_254 _coated plates (Merk) using the following solvent systems: DCM-MeOH (9:1 and 4:1).

**Animals**

Swiss albino rats (170 - 180 g) and mice (25 - 30 g) were obtained from the animal house of the Department of Pharmacology and Toxicology, Nnamdi Azikiwe University, Awka. The animals were housed under standard laboratory conditions and fed with rodent feed (Guinea Feeds Nigeria Ltd). They had free access to food and water. All animal experiments were conducted in compliance with NIH guide for care and use of laboratory animals (Pub No: 85-23 Revised 1985) as well as with our Institutional ethical guidelines for use of laboratory animals.

**Acute toxicity study**

Acute toxicity test was conducted according to OECD guidelines (OECD, 2011 ▶). A limit dose of 2000 mg/kg was used to determine the acute toxicity effect of the extract. A total of 3 mice and rats was administered with2000 mg/kg of the extract and observed closely for signs of toxicity and mortality for 24 hr and thereafter for a total of 14 days.

**CCl**_4_ **-induced oxidative stress**

The effect of the extract and fractions on CCl_4_-induced oxidative stress was conducted using 48 albino rats divided into 8 groups of 6 animals each. The extract and fractions were administered orally at the doses of 200 and 400 mg/kg for 10 days. Alpha-tocopherol (60 mg/kg) served as the standard while the control groups (normal control and CCl_4_-induced control) received 10 ml/kg tween 20. Oxidative stress was induced using single dose of CCl_4_ 6 hr after the last treatment. On the 11^th^ day, blood samples were collected from the animals through retro-orbital plexus for determination of antioxidant enzymes (Catalase and Superoxide dismutase) and lipid peroxidation. 

**Determination of superoxide dismutase (SOD) activity**

Superoxide dismutase activity was determined by its ability to inhibit the auto-oxidation of epinephrine determined by the increase in absorbance at 480 nm as described by Sun and Zigma (1978) ▶. An extinction coefficient for epinephrine at 480 nm of 4020 M^-1^cm^-1^ was used in calculating activity.

**Determination of catalase (CAT) activity**

Catalase activity was determined according to the method of Beers and Sizer as described by Usoh et al., (2005). An extinction coefficient for H2O2 at 240 nm of 40.0 M-1cm-1 as described by Aebi (1984) ▶ was used for the calculation.

**Lipid peroxidation (LPO) assay**

Malondialdehyde (MDA), an index of lipid peroxidation, was determined using the method described by Ogbunugafor et al. (2012) ▶ with little modification. Here, 1.0 ml of the sample was added to 2 ml of (1:1:1) TCA-TBA-HCL-reagent (15% TCA, thiobarbituric acid 0.37% and 0.24 N HCl). The mixture was boiled at 100 ^o^C for 15 min, allowed to cool and then, centrifuged at 3000 rpm for 10 min. The supernatant was measured using spectrophotometer at 532 nm against a blank. MDA concentration was calculated using the molar extinction coefficient for MDA TBA-complex of 1.56 × 10^5 ^M^-1 ^cm^-1^.

**Hydrogen peroxide scavenging activity**

Hydrogen peroxide scavenging activity of the isolated compounds was determined using the method of Kumaran and Karunakaran (2007) ▶ with little modification. A solution of hydrogen peroxide (20 mM) was prepared in phosphate buffer saline (pH 7.4). Here, 2 ml of this solution was added to 1 ml of different concentrations of test compounds (10, 20, 40, 60, 80 and 100 µg/ml) and absorbance of hydrogen peroxide content of the mixture was read at 230 nm after 10 min against a blank solution containing phosphate buffer without hydrogen peroxide. Ascorbic acid served as the standard. The percentage of scavenging potential was calculated using the following equation:

Hydrogen peroxide scavenging activity (%) = 1-Abs of control - Abs of sampleAbs of control x 100

**DPPH free radical scavenging activity test**

The DPPH free radical scavenging activity of the isolated compounds was evaluated based on the method described by Patel and Patel (2010) ▶ with modification. Here, 25 µl of freshly prepared DPPH solution (0.6 mmol) was added to 25 µl of different concentrations of the test compounds (10, 20, 40, 60, 80 and 100 µg/ml). The volume of the solution was adjusted with methanol to a final volume of 200 µl. The control tube contains 175 µl methanol and 25 µl of DPPH. After incubation in the dark for 30 min at room temperature, absorbance of the mixtures was measured at 490 nm using micro plate reader. All the tests were performed in triplicate and ascorbic acid was used as standard. The percentage of DPPH radical scavenging potential of the compounds and standard control (ascorbic acid) were calculated from the equation below.

 % Inhibition of free radical = [(A_o_–A_1_) / A_o_] × 100 

Where A_o_ is the absorbance of the control, and A_1_ is the absorbance of the test compound/standard. The EC_50 _was also determined from percentage of scavenging potential. 

**Ferric-reducing antioxidant power (FRAP) assay**

Different concentrations of each of the isolated compounds (10, 20, 40, 60, 80 and 100 µg/ml) in 0.5 ml of methanol were mixed with phosphate buffer (1.25 ml, 0.2 M, pH 6.6) and potassium ferricyanide [K3Fe(CN)6] (1.25 ml, 1%). The mixture was incubated at 50° C for 20 min. Then, 1.25 ml of 10% tricholoroacetic acid was added to the mixture and centrifuged at 3,000 rpm for 10 min at room temperature. The upper layer of solution (1.25 ml) was mixed with distilled water (1.25 ml) and ferric chloride (FeCl_3_) (0.25 ml, 0.1%) and the absorbance of the reaction mixture was measured at 700 nm (Kumar, 2005 ▶). Percentage of reducing potential was determined. 

**Statistical analysis**

Results obtained were analyzed using SPSS version 18.0 and results were expressed as mean + SEM. Differences among means was considered significant at p<0.05. The graphs were plotted using MS Excel with the average of three observations. Antioxidant activity of the test compounds were quantified using EC_50_.

## Results

At 2000 mg/kg limit dose acute toxicity test, no mortality or obvious sign of toxicity was observed in both animal species (rats and mice). 

**Figure 1:**
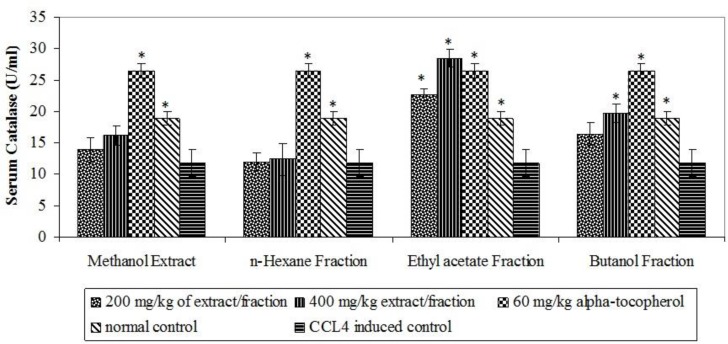
Effect of extract and fractions on serum catalase enzymes. * p< 0.05 compared with CCl_4 _induced control.

**Figure 2:**
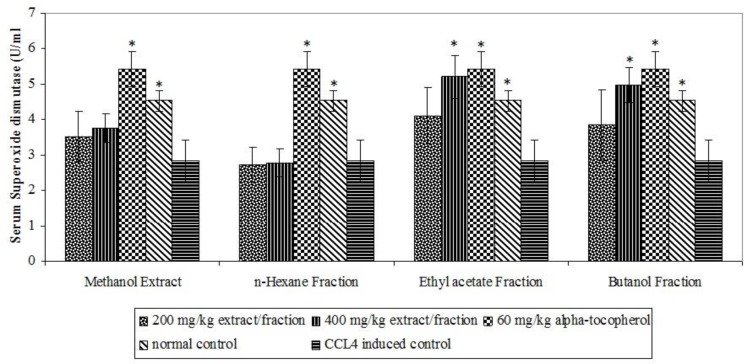
Effect of extract and fractions on serum superoxide dismutase enzyme. * p< 0.05 compared with CCl_4 _induced control.

**Figure 3:**
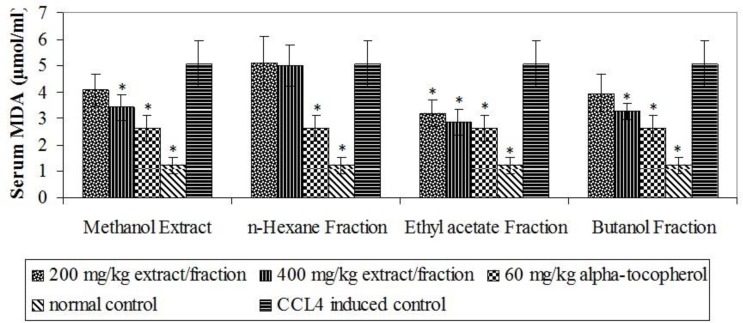
Effect of extract and fractions on serum malondialdehyde. * p< 0.05 compared with CCl_4 _induced control.

The oxidative stress inductive potentials of CCl_4_ were confirmed from significant (p<0.05) elevation of MDA (a product of lipid peroxidation) and reduction in antioxidant defence enzymes in CCl_4_-induced control (Figures 1, 2 and 3). The ethyl acetate fraction stimulated significant (p<0.05) elevation of SOD enzyme at 400 mg/kg and CAT at both 200 and 400 mg/kg while butanol fraction produced significant (p<0.05) elevation of both enzymes at 400 mg/kg (Figures 1 and 2).

**Table 1:** Qualitative phytocompounds of the extract and fractions

Phytocompounds	Ethanol extract	n-Hexane fraction	Ethyl acetate fraction	Butanol fraction
Alkaloids	**++**	**-**	**-**	**++**
Saponins	**++**	**-**	**-**	**++**
Flavonoids	**+++**	**-**	**+++**	**++**
Glycosides	**++**	**-**	**-**	**+++**
Terpenoids	**+++**	**-**	**++**	**+**
Steroids	**+++**	**+++**	**-**	**-**
Resins	**-**	**-**	**-**	**-**
Tannins	**+**	**-**	**+**	**-**

- = absent, + = present at low concentration, ++ present at moderately high concentration, +++ = present at high concentration.

Preliminary phytochemical investigation showed the presence of diverse phytocompounds. Steroids were partitioned in the n-hexane fraction while phenolic compounds, particularly flavonoids, were abundant in the ethyl acetate fraction (Table 1). Ethyl acetate fraction also showed the highest content of phenolic compounds with total phenolic content of 319.4 mg GAE/g compared to the extract, n-hexane and butanol fractions that had 140.1, 8.9 and 248.5 mg GAE/g, respectively (Table 2). 

**Table 2:** Total phenolic content of the extract and fractions

Samples	Mean Gallic acid Equivalent (mg GAE/g)
Methanol Extract	140.1 + 0.7
n-Hexane Fraction	8.9 + 0.9
Ethyl acetate Fraction	319.4 + 0.5
Butanol Fraction	248.5 + 0.6

**Figure 4:**
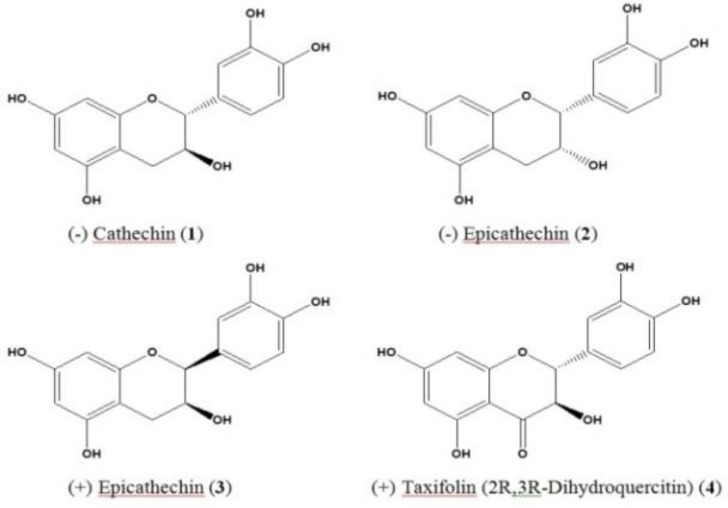
Chemical structures of the isolated compounds.

Further purification of the ethyl acetate fraction led to isolation of four known compounds. The chemical structures of these compounds were elucidated as the flavans (-) cathechin (1), (-) epicathechin (2), (+) epicathechin (3) and the flavanone, (+) taxifolin (2R, 3R dihydroquercitin) (4) (Figure 4) based on the analysis of their 1- and 2-D NMR and mass spectrometry and comparison of data (Table 3) with those previously reported in the literature (Nonaka et al., 1987 ▶). 

**Table 3:** 1H NMR data of (-) cathechin (1), (-) epicathechin (2), (+) epicathechin (3) and (+) taxifolin (4).

**Position **	***δ***_H _ **(1)**	***δ***_H _ **(2)**	***δ***_H_ ** (3) **	***δ***_H_ ** (4)**
**1**	**-**	**-**	**-**	**-**
**2**	**4.50 d (7.5)**	**4.82 brs**	**4.81 brs**	**4.90 d (11.4)**
**3**	**3.96 ddd(7.5, 5.4, 8.1) **	**4.18 m**	**4.18 m**	**4.49 d (11.4)**
**4**	**2.85 dd (5.4, 16.1) Ha** **2.86 dd (4.4, 16.7)Ha **	**2.85 dd (4.5, 16.8) Ha - **	**2.50 dd (8.1, 16.1) ** **Hb2.74 dd (16.7) Hb **	**2.74 d (16.8) **
**5**	**-**	**-**	**-**	**-**
**6**	**5.92 d (2.3) **	**5.94 d (2.3)**	**5.94 brs **	**5.90 brs**
**7**	**-**	**-**	**-**	**-**
**8**	**5.85 d (2.3) **	**5.91 d (2.2) **	**5.91 brs**	**5.80 brs**
**9**	**-**	**-**	**-**	**-**
**10**	**-**	**-**	**-**	**-**
**1'**	**-**	**-**	**-**	**-**
**2'**	**6.84 d (1.8) **	**6.97 d (1.8)**	**6,97 brs **	**6.97 (1.9)**
**3'**	**-**	**-**	**-**	**-**
**4'**	**-**	**-**	**-**	**-**
**5'**	**6.76 d (8.1) **	**6.76 d (8.1)**	**6.76 d (8.1)**	**6.80 d (8.1)**
**6'**	**6.72 dd (1.8, 8.1) **	**6.79 dd (1.8, 8.1) **	**6.80 d (8.2)**	**6.84 dd (1.9, 8.1)**

(-) Cathechin (1): Light brown solid (10.0 mg); UV: _ max_ (PDA) = 280.0 nm. ESIMS (+ve): m/z = 291.1 [M+H]^+^, 580.9 [2M+1]^+^, 602.9 [2M+Na]^+^ ESIMS (-ve): m/z = 289.3 [M-H]^-^. ^1^H NMR (MeOH-d_4_) (Table 3).

(-) Epicathechin (2): Light brown solid (10.0 mg); UV: _ max_ (PDA) = 280.0 nm. ESIMS (+ve): m/z = 291.1 [M+H]^+^, 580.9 [2M+1]^+^, 602.9 [2M+Na]^+^ ESIMS (-ve): m/z = 289.3 [M-H]^-^. ^1^H NMR (MeOH-d_4_) (Table 3).

(+) Epicathechin (3): Light brown solid (11.0 mg); UV: _ max_ (PDA) = 280.0 nm. ESIMS (+ve): m/z = 291.1 [M+H]^+^, 580.9 [2M+1]^+^, 602.9 [2M+Na]^+^ ESIMS (-ve): m/z = 289.3 [M-H]^-^. ^1^H NMR (MeOH-d_4_), (Table 3).

(+) Taxifolin (4): Light brown solid (12.0 mg); UV: _ max_ (PDA) = 290.0 nm; ESIMS (+ve): m/z = 305.1 [M+H]^+^, 327.0 [M+Na]^+^, 630.8 [2M+Na]^+^; ESIMS (-ve): m/z = 303.2 [M-H]^-^, 606.8 [2M-H]^-^; ^1^H NMR (MeOH-d_4_) (Table 3).

**Figure 5:**
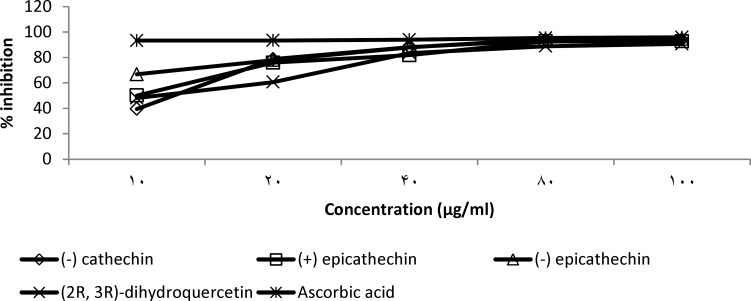
Hydrogen peroxide scavenging activity of test compounds

The significant (p< 0.05) elevation of CAT enzyme observed with the ethyl acetate fraction was found to be complemented by *in vitro* hydrogen peroxide scavenging activity of the flavans and flavanone isolated from this fraction (Figure 5). It could be seen in Figure 5 that the isolated flavonoid compounds showed effective scavenging of H_2_O_2_ at lower concentrations. (-) epicathechin showed the same EC_50_ of 8 µg/ml as ascorbic acid while (-) cathechin, (+) cathechin and 2R, 3R dihydroquercitin showed EC_50_ values of 13, 10 and 18 μg/ml, respectively (Table 4). The ethyl acetate fraction at 200 and 400 mg/kg produced significant (p< 0.05) reduction of CCl_4_-induced lipid peroxidation while significant (p< 0.05) reduction by the extract and butanol fraction was noticed at 400 mg/kg (Figure 3). 

**Figure 6:**
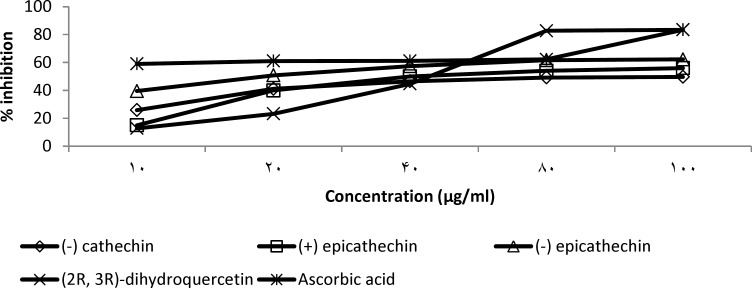
DPPH scavenging activity of test compounds

The DPPH scavenging activity of the isolated flavonoid compounds showed a dose-response relationship (Figure 6). 2R, 3R dihydroquercitin at 100 μg/ml produced the same scavenging efficacy as ascorbic acid with 84.3% inhibition. However, ascorbic acid exhibited better activity at lower concentration, having an EC_50_ of 6 µg/ml against 46 μg/ml recorded for 2R, 3R dihydroquercitin (Table 4). Also, (-) epicathechin and (+) epicathechin exhibited better scavenging activity at lower concentration with an EC_50_ values of 19 and 40 µg/ml, respectively. The least activity was recorded for (-) cathechin with an EC_50_ value of 88 μg/ml.

**Figure 7:**
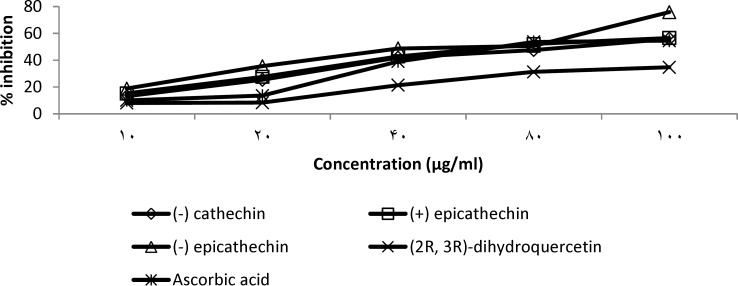
Ferric reducing antioxidant power test of compounds

For the ferric reducing antioxidant power (FRAP) assay, (-) epicathechin exhibited higher reducing efficacy with EC_50_ of 46 μg/ml compared to ascorbic acid (66 μg/ml) (Figure 7 and Table 4). The reducing ability of (+) epicathechin was similar to ascorbic acid with an EC_50_ of 68 µg/ml while the EC_50_ value of (-) cathechin was 88 μg/ml. At the tested doses, 2R, 3R dihydroquercitin did not produce up to 50% reduction of ferric ion, showing only 34.7% inhibition at 100 μg/ml.

**Table 4:** EC_50_ values of isolated compounds in *in vitro* antioxidant models

In vitro models	EC_50 _(µg/ml)				
	(-) cathechin	(+) epicathechin	(-) epicathechin	(2R, 3R) - dihydroquercetin	Ascorbic acid
DPPH	88	40	19	46	6
FRAP	88	68	46	-	66
H_2_O_2_	13	10	8	18	8

## Discussion

Oxidative stress has been implicated in many disease states including cancer, inflammation, Azheimer’s and other neurodegenerative disorders (Soobratte et al., 2006 ▶; Jones, 2008 ▶). Reduction of oxidative stress has thus been found to contribute to the maintenance of healthy living (Hamilton et al., 2012 ▶). Herbal anti-oxidants are bioactive phytoconstituents with the ability to scavenge reactive oxygen and nitrogen species, which are the major causes of oxidative stress in the body (Burton and Jauniaux 2011 ▶). The use of natural antioxidant in the management of disorders associated with oxidative stress is becoming an attractive alternative in recent time (Vijay and Vimukta 2014). In the present study, we have investigated both the in vitro and in vivo antioxidant potential of a medicinal plant, *Alchornea floribunda*, which has been widely used in the management of inflammation, arthritis, and wound infection.

No incidence of mortality and obvious signs of toxicity observed in acute toxicity study is an indication of the likely oral acute safety of the extract. Then, we investigated the extracts and their solvent fractions for their ability to ameliorate CCl_4_-induced oxidative stress. Carbon tetrachloride (CCl_4_) is a known inducer of oxidative stress which acts through its hepatic metabolites (Said and Husein, 2009). Superoxide dismutase enzyme (SOD) and Catalase (CAT) form important enzymatic defence against oxidative stress. SOD converts superoxide radical to hydrogen peroxide which is inactivated by CAT to water (Thirunavakkarasu et al., 201 ▶0). Elevation of this complementary enzymatic defence system by the ethyl acetate and butanol fractions may have contributed to neutralization of the possible deleterious consequences of CCL_4_-induced oxidative stress. Enzyme induction by phenolic compounds has been demonstrated by Yeh and Yen (2006). The abundance of phenolic compounds in the most active fractions may account for their good antioxidant activity. Our results thus corroborated the beneficial roles of phenolic compounds in free radical-mediated processes, which have been previously established (Cartea et al., 2011 ▶). 

 The observed *in vivo* activity of the extracts and fractions was thus corroborated by the *in vitro* antioxidant studies of the isolated flavonoids as demonstrated, in particular, through their hydrogen peroxide scavenging ability. This demonstrates that these flavonoid compounds may not only induce the production of hydrogen peroxide (H_2_O_2_) scavenging enzyme but also have direct scavenging effect on circulating H_2_O_2_. Biologically, although H_2_O_2 _is widespread and poorly reactive, it is capable of direct inactivation of sulfhydryl (–SH) group containing enzymes (Halliwell and Gutteridge, 2007 ▶). The toxicity of H_2_O_2 _is mainly due to its ability to produce highly reactive hydroxyl radical through the Fenton reaction (Thomas et al., 2009 ▶). The ability of the isolated compounds to scavenge H2O2 reflects their likelihood to inhibit formation of hydroxyl radical from H_2_O_2_. The scavenging of H_2_O_2_ by the compounds demonstrated their hydrogen atom donating ability, a common feature of most flavonoids compound (Dragan et al., 2007 ▶). 

Oxidative degradation of unsaturated lipids is another deteriorating effect of free radicals that leads to lipid peroxidation (Barrera, 2012 ▶). The high content of unsaturated fatty acids in biological membranes makes them most susceptible to free radical-induced oxidative stress (Nowak, 2013 ▶). MDA is a key product of lipid peroxidation and as such, a very sensitive indicator of lipid peroxidation (Pandey et al., 2012 ▶). Phenolic compounds in addition to their ability to neutralize all types of oxidizing radicals also act as powerful chain breaking antioxidants (Khalili et al., 2013 ▶). This characteristic accounts for the role of phenolic compounds in mediating membrane-dependent processes such as free radical-induced membrane lipid peroxidation (Veeru et al., 2009 ▶). Their structural characteristics and their ability to interact with membrane phospholipids and penetrate lipid bilayers make them effective membrane stabilizers and inhibitors of lipid peroxidation (Michalak, 2006 ▶; Veeru et al., 2009 ▶). Electron and/or hydrogen donation contributes to chain braking process, an important step in inhibition of lipid peroxidation (Lobo et al., 2010 ▶). The electron and/or hydrogen donating abilities of the phenolic content of ethyl acetate fraction was demonstrated by the *in vitro* DPPH and FRAP tests of the flavans and flavanone isolated from this fraction. 

The observed DPPH scavenging activity of the isolated flavonoid compounds is in line with numerous studies on scavenging properties of flavonoids (Okawa et al., 2001 ▶; Zhang et al., 2012 ▶) isolated from medicinal plants. Cathechin and epicatechin isolated from red grape were shown by Iacopini et al (2008) ▶ to possess strong anti-radical activity and dihydroquercitin isolated from *Larix gmelinii* has been shown to demonstrate effective antioxidant activity (Kolhir et al., 2006 ▶). Of particular importance is the hydrogen (electron) donating ability of flavonoids which acts to scavenge reactive species (Dragan et al., 2007 ▶). This ability is in part attributed to the 3', 4' ortho hydroxylated functional groups (Dragan et al., 2007 ▶) and this might have contributed to the activity exhibited by the isolated flavonoid compounds. 

The FRAP mechanism is totally single electron transfer unlike DPPH test that combines hydrogen atom transfer with single electron transfer (Huang et al., 2005 ▶). It may explain why 2R, 3R dihydroquercitin exhibited higher hydrogen atom transfer-mediated scavenging of free radical than single electron transfer and thus could explain the lower activity recorded by this compound for FRAP assay. Also, some phenolic compounds have been shown to react more slowly in FRAP assay (Ronald et al., 2005 ▶).

 Although we are much encouraged by the apparent antioxidant properties of *A. floribunda* in our animal models, further study on longer term toxic effects on various organs are needed before the use of the drug in humans is justified. 
